# Phenethylferulate as a natural inhibitor of inflammation in LPS-stimulated RAW 264.7 macrophages: focus on NF-κB, Akt and MAPK signaling pathways

**DOI:** 10.1186/s12906-023-04234-y

**Published:** 2023-11-07

**Authors:** Zhongjie Yan, Yuanyu Wang, Yizhen Song, Yicong Ma, Yufan An, Ran Wen, Na Wang, Yun Huang, Xiuwen Wu

**Affiliations:** 1 Department of Neurosurgery, the Second Hospital of Hebei Medical University, Shijiazhuang, Hebei 050000 China; 2 School of Pharmaceutical Sciences, Hebei Medical University, Shijiazhuang, Hebei 050017 China

**Keywords:** Phenethylferulate, Anti-inflammation, LPS-stimulated RAW 264.7 macrophages, NF-κB, Akt, MAPK

## Abstract

**Background:**

*Notopterygii Rhizoma et Radix* (NRR) is commonly used for the treatment of inflammation-linked diseases. Phenethylferulate (PF) is high content in NRR crude, but its anti-inflammatory effect remains unclear. Therefore, we aimed to investigate the anti-inflammatory properties of PF and its underlying molecular mechanisms in lipopolysaccharide (LPS)-stimulated RAW 264.7 macrophages.

**Methods:**

The effect of PF on cell viability was measured by MTT assay. The anti-inflammatory properties of PF were studied by detecting the levels of inflammatory mediators and cytokines using enzyme-linked immunosorbent assay (ELISA). Furthermore, the anti-inflammatory mechanisms of PF were determined by Western blot analysis.

**Results:**

PF was not cytotoxic to RAW 264.7 macrophages at the concentrations of below 48 μM. ELISA showed that PF conspicuously inhibited overproduction of prostaglandin E_2_ (PGE_2_), tumor necrosis factor α (TNF-α), interleukin 1β (IL-1β) and interleukin 6 (IL-6). Western blot analysis showed that PF remarkably suppressed overproduction of inducible nitric oxide synthase (iNOS) and cyclooxygenase 2 (COX-2), the phosphorylation of inhibitor of NF-κB kinase α (IκB-α), protein kinase B (Akt), extracellular signal-regulated kinase (ERK), c-Jun N-terminal kinases (JNK) and p38, as well as the degradation and subsequent nuclear translocation of p65.

**Conclusions:**

PF is a potent inhibitor of inflammation acting on nuclear factor kappa-B (NF-κB), Akt and mitogen-activated protein kinase (MAPK) signaling pathways in LPS-stimulated RAW 264.7 macrophages. This work provides evidence for the suitability of PF as a therapeutic candidate for the management of inflammatory-mediated immune disorders.

**Supplementary Information:**

The online version contains supplementary material available at 10.1186/s12906-023-04234-y.

## Background

Inflammation is a part of the body’s natural immune response to microbial invasion, but excessive inflammation can cause tissue damage and diseases [1]. Macrophages, as important immune cells, play an indispensable role in the first line of defense against invaders. In the process of pathologic inflammation, macrophages produce excessive inflammatory mediators, which then trigger a series of inflammatory reactions [2]. Pathologic inflammation is associated with many diseases, and thus controlling inflammation is an important goal of preventing and treating inflammatory diseases [3].

Lipopolysaccharide (LPS), a cell wall component of Gram-negative bacteria, can stimulate macrophages via Toll-like receptors (TLRs), especially TLR4, leading to the activation of nuclear factor kappa-B (NF-κB), protein kinase B (Akt), mitogen-activated protein kinase (MAPK) and other inflammatory signaling pathways, which then lead to release of excessive inflammatory mediators and cytokines, such as nitric oxide (NO), prostaglandin E_2_ (PGE_2_), tumor necrosis factor α (TNF-α), interleukin 1β (IL-1β), interleukin 6 (IL-6), interleukin 10 (IL-10), and subsequently trigger a series of abnormal inflammatory response [4–9]. LPS-stimulated RAW264.7 cell inflammation model is a popular and mature model for investigating anti-inflammatory activity [10, 11]. As reported, the therapeutic effects of some phenolic acids (e.g. caffeic acid phenethyl ester, ethyl ferulate, resveratrol and curcumin) in LPS-stimulated RAW264.7 cell model were related to their inhibition of TLR4 signaling pathway and overexpression of downstream signaling proteins [12–15].

*Notopterygii Rhizoma et Radix* (NRR), an important constituent of traditional Chinese medicine, has a long history of medicinal application. In clinical practice, NRR has a good therapeutic effect on inflammation-linked diseases, such as rheumatic arthritis, rheumatoid arthritis, infectious pneumonia, bronchitis, etc. [16]. In the preliminary screening of active constituents of NRR, we obtained a series of phenolic acid compounds with anti-inflammatory activity [17]; among them, phenethylferulate (PF), high content in the crude, was a potently efficiency ingredient [18]. At present, there is a lack of in-depth research on the mechanisms of anti-inflammatory role of PF, which limits its further development and utilization.

Therefore, in the current study, we explored the anti-inflammatory properties of PF by detecting the release of inflammatory mediators and cytokines and its potential molecular mechanisms by monitoring the expression of NF-κB, Akt and MAPK signaling pathway related proteins in LPS-stimulated RAW264.7 cell inflammation model.

## Materials and methods

### Materials

PF was isolated and purified from NRR, and identified by various spectroscopic methods in our lab [17], with a purity of 98.1% by high performance liquid chromatography (HPLC). Dulbecco’s modified eagle’s medium (DMEM), fetal bovine serum (FBS), phosphate buffered saline (PBS), and trypsin were purchased from Gibco® Laboratories (Life Technologies Inc., Grand Island, NY, USA). Penicillin-streptomycin solution, bovine serum albumin(BSA), nuclear and cytoplasmic extraction reagents and enhanced chemilu-minescent (ECL) kit were obtained from Suolaibao Technology Ltd. (Beijing, CN). 3-(4,5-Dimethylthiazol-2-yl)-2,5-diphenyltetrazolium bromide (MTT), dimethyl sulfoxide (DMSO) and LPS were supplied by Sigma-Aldrich Co. (St. Louis, MO, USA). Murine enzyme-linked immunosorbent assay (ELISA) kits for PGE_2_, TNF-α, IL-1β, IL-6, and IL-10 were acquired from R&D Systems (St. Louis, MO, USA). Bicinchoninic acid (BCA) kit and radioimmunoprecipitation assay (RIPA) lysis buffer were purchased from Beyotime Biotechnology (Haimen, Jiangsu, CN). Rabbit monoclonal antibodies for inducible nitric oxide synthase (iNOS), cyclooxygenase 2 (COX-2), inhibitor of NF-κB kinase α (IκB-α)/p-IκB-α, p65, Akt/p-Akt, extracellular signal-regulated kinase (ERK)/p-ERK, c-Jun N-terminal kinases (JNK)/p-JNK and p38/p-p38 were purchased from Abcam (Cambridge, MA, USA). β-actin and Lamin-B1 were purchased from Wuhan Servicebio Technology (Wuhan, Hubei, CN).

### Methods

#### Cell culture

The murine macrophage cell line RAW264.7 cell (3111C0001CCC000146) was obtained from the Cell Resource Center, Peking Union Medical College (Beijing, CN). Cells were maintained in DMEM containing 10% FBS, 50 units/ml of penicillin and 50 μg/ml of streptomycin, in a constant humidity atmosphere of 5% CO_2_ and 95% air at 37 °C.

#### Cell viability assay

MTT assay was used to assess the effect of PF on cell viability. RAW 264.7 cells (5 × 10^3^ cells/well) were seeded into 96-well plates for 24 h in a constant humidity atmosphere of 5% CO_2_ and 95% air at 37 °C. The cells were then exposed to PF (0, 3, 6, 12, 24 and 48 μM) for 24 h with or without LPS (1 μg/mL), and followed by the addition of MTT solution (0.2 mg/mL). After 4 h incubation, the medium was removed, and the formazan crystals in each well were dissolved in DMSO. The absorbance of each well was measured at 570 nm using a microplate reader.

#### Determination of inflammatory mediators and cytokines in culture supernatant

ELISA was used to determine the inhibitory effects of PF on the production of inflammatory mediators and cytokines in LPS-stimulated RAW 264.7 macrophages. Briefly, RAW 264.7 cells (3 × 10^5^ cells/well) were seeded into 24-well plates and preincubated with PF (0, 3, 6, and 12 μM) for 1 h, followed by the addition of LPS (1 μg/mL) for 24 h. Non-LPS-treated group was regarded as control. Culture supernatants were collected and the release of PGE_2_, TNF-α, IL-1β, IL-6 and IL-10 were evaluated using ELISA kits according to the manufacturer’s instructions.

#### Western blot

Western blot was used to measure the relevant proteins expression. In brief, RAW 264.7 cells were seeded and pretreated with PF (0, 3, 6, and 12 μM) for 1 h, followed by the addition of LPS (1 μg/mL) for 24 h. Afterwards, the cells were collected and lysed in RIPA lysis buffer to obtain the total protein. Nuclear and cytosolic extracts of collected cells were fractionated using nuclear and cytoplasmic extraction reagents according to the manufacturer’s protocol. The protein concentration was measured using BCA protein quantitation kit. An equal amount of protein (30 μg) was electrophoresed through 10% SDS-PAGE gels and separated onto polyvinylidene fluoride (PVDF) membranes, which were then blocked by 5% BSA solution for 1 h at room temperature and then incubated with different primary antibodies over night at 4 °C. Next, the PVDF membranes were incubated with corresponding secondary antibodies for 2 h at room temperature. The primary antibodies were as follows: iNOS, COX-2, IκB-α/p-IκB-α, p65, Akt/p-Akt, ERK/p-ERK, JNK/p-JNK, p38/p-p38. Finally, the expressions of proteins were detected by an ECL kit. Band signal intensities were measured by densitometry and quantified using the Image J Software. Non-LPS-treated group was regarded as control. Data for cytoplasmic proteins and nuclear proteins were normalized to the levels of β-actin and Lamin-B1 respectively, and expressed relative to the control.

#### Statistical analysis

Data were analyzed in SPSS 17.0 software and expressed as mean ± SD from at least three independent experiments. Differences among different groups were assessed using Student’s t-text and one-way ANOVA. Statistical significance was defined as *p* < 0.05.

## Results

### Cytotoxicity of PF on RAW264.7 macrophages

**Fig. 1:**
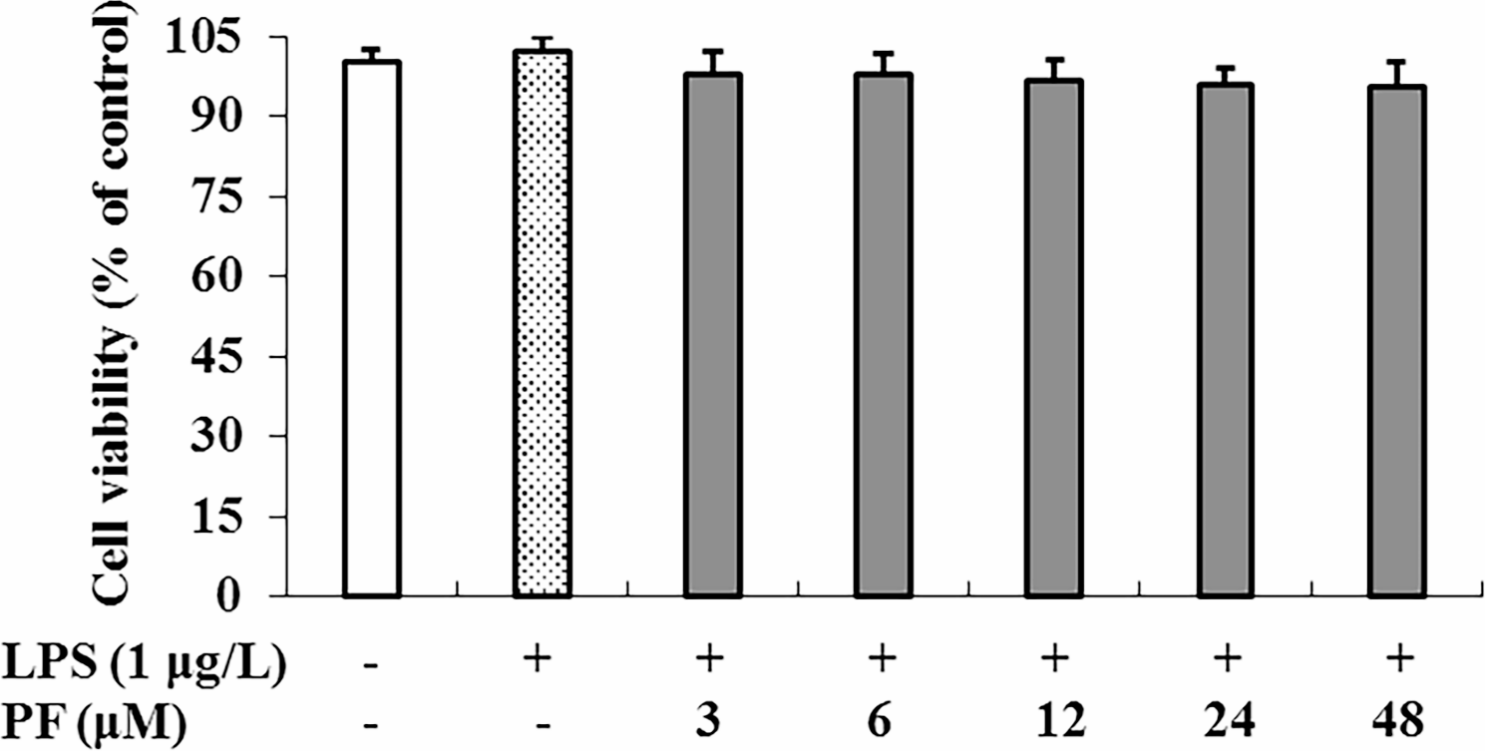
Cytotoxicity of PF on RAW 264.7 macrophages. Cell viability was measured by the MTT assay after 24 h treatment of PF (0, 3, 6, 12, 24 and 48 μM) in the presence of LPS (1 μg/mL). Neither LPS nor PF-treated group was selected as control, and expressed as 100%. Data are expressed as mean ± SD from three independent experiments

MTT assay showed that cell viability was unaffected after 24 h treatment with PF at different concentrations (0, 3, 6, 12, 24 and 48 μM) in the presence or absence of 1 μg/ml of LPS (Fig. 1). Therefore, PF were considered to have no cytotoxicity on RAW264.7 cells at the concentrations of below 48 μM, which was consistent with the previous experiment [17]. Based on the non-toxic dose range and the previous result that PF had an inhibitory activity on NO overproduction in LPS-activated RAW264.7 macrophage cell model with IC_50_ value of (2.73 ± 0.58) μM, the low concentration was set to 3 μM, and so on, the middle concentration was set to 6 μM, and the high concentration was set to 12 μM in subsequent experiments.

### Inhibition of PF on LPS-stimulated PGE_2_ production in RAW 264.7 macrophages

**Fig. 2:**
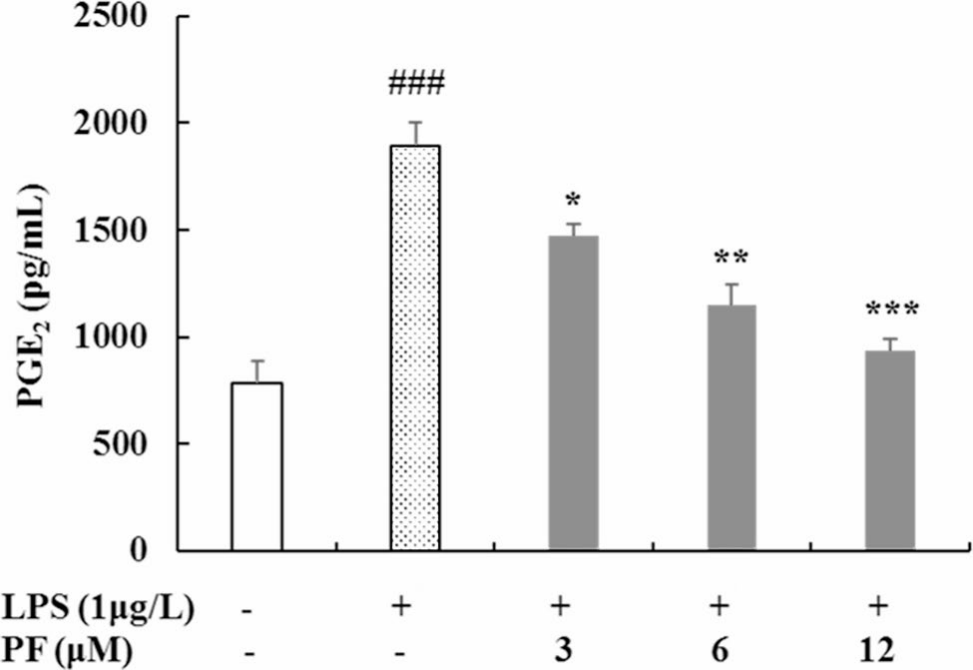
Effects of PF on the production of PGE_2_ in LPS-stimulated RAW 264.7 macropages. RAW 264.7 cells were pretreated with PF (0, 3, 6, and 12 μM) for 1 h, followed by the addition of LPS (1 μg/mL) for 24 h. The supernatant was collected for the level of PGE_2_ using ELISA kits. Neither LPS nor PF-treated group was selected as control. Data are expressed as mean ± SD from three independent experiments. ### *p* < 0.001 compared with control group; * *p* < 0.05, ** *p* < 0.01, *** *p* < 0.001 compared with LPS alone-treated group

To clarify the potential anti-inflammatory effect of PF, PGE_2_ release were also measured in LPS-stimulated RAW 264.7 macrophages. PF was found to dose-dependently inhibit the overproduction of PGE_2_ activated by LPS in RAW 264.7 macrophages (Fig. 2). In particular, 12 μM of PF, which was in the highest treatment concentration, dramatically decreased PGE_2_ expression levels by 50.5% compared with the LPS alone-activated group (*P* < 0.001).

### Inhibition of PF on LPS-stimulated TNF-α, IL-1β and IL-6 production in RAW 264.7 macrophages

**Fig. 3:**
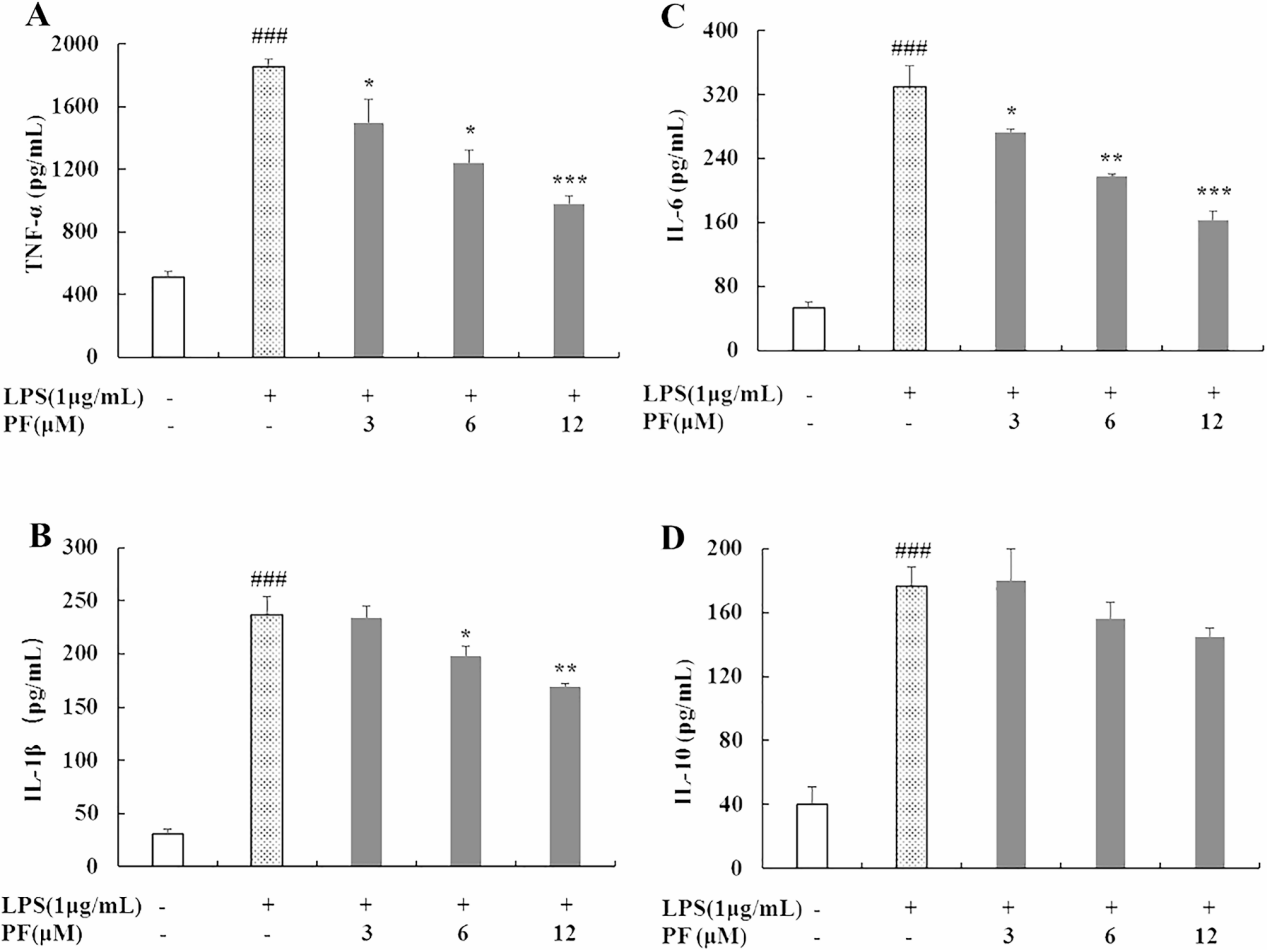
Effects of PF on the production of TNF-α (**A**), IL-1β (**B**), IL-6 (**C**), and IL-10 (**D**) in LPS-stimulated RAW 264.7 macropages. RAW 264.7 cells were pretreated with PF (0, 3, 6, and 12 μM) for 1 h, followed by the addition of LPS (1 μg/mL) for 24 h. The supernatant was collected for the levels of TNF-α (**A**), IL-1β (**B**), IL-6 (**C**), and IL-10 (**D**) using ELISA kits. Neither LPS nor PF-treated group was selected as control. Data are expressed as mean ± SD from three independent experiments. ### *p* < 0.001 compared with control group; * *p* < 0.05, ** *p* < 0.01, *** *p* < 0.001 compared with LPS alone-treated group

Since PF was found to potently inhibit the release of NO and PGE_2_ in LPS-stimulated RAW264.7 macrophages, its effects on LPS-stimulated overproduction of inflammatory mediators and cytokines were further investigated. The investigated indicators included TNF-α, IL-1β, IL-6 and IL-10, and the results are summarized in Fig. 3. As shown in Fig. 3A, B and C, administration of PF dose-dependently inhibited LPS-stimulated TNF-α, IL-1β and IL-6 overproduction in contrast with LPS alone-stimulated group, with the exception that 3 μM of PF had no influence on the secretion of IL-1β. Notablely, PF at the highest dose concentration markedly decreased TNF-α, IL-1β and IL-6 expression levels by 47.2%, 28.4%, 50.2%, compared with those treated with LPS alone (*P* < 0.001, *P* < 0.01, *P* < 0.001). In our test, treatment with PF (3 μM, 6 μM and 12 μM) non-significantly suppressed IL-10 levels in LPS-stimulated RAW 264.7 macrophages (Fig. 3D).

### Suppression of PF on LPS-stimulated iNOS and COX-2 protein expression

**Fig. 4:**
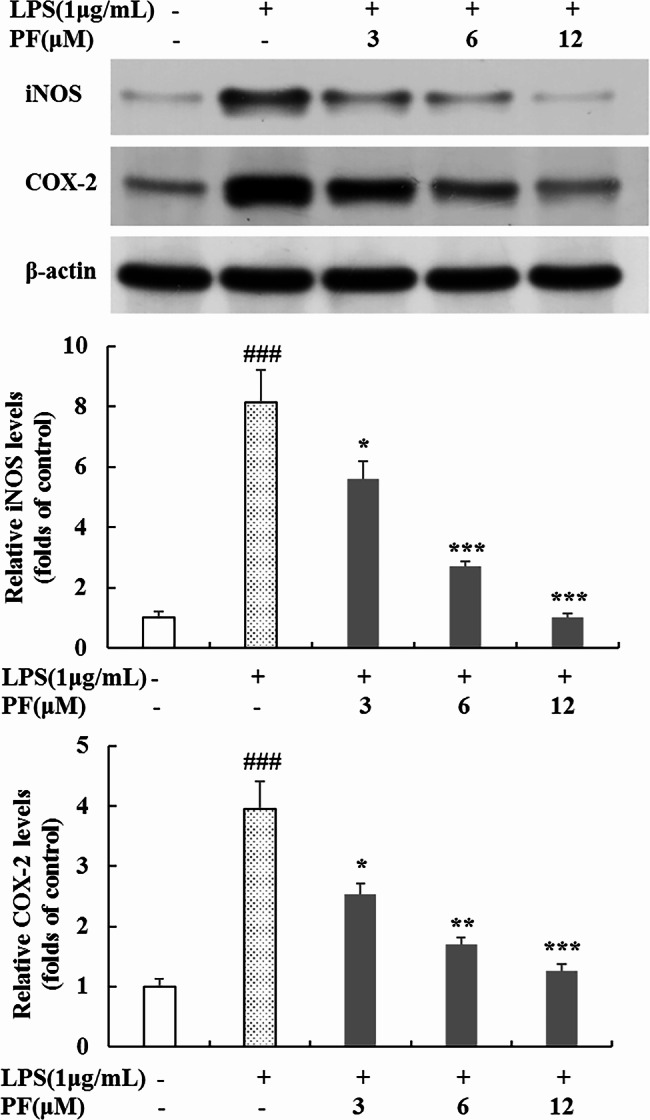
Effects of PF on iNOS and COX-2 protein expressions in LPS-stimulated RAW 264.7 macropages. RAW 264.7 cells were pretreated with PF (0, 3, 6, and 12 μM) for 1 h, followed by the addition of LPS (1 μg/mL) for 24 h. The protein levels of iNOS and COX-2 in cytoplasmic proteins were examined by Western blot analysis. β-actin was used as a loading control. Neither LPS nor PF-treated group was selected as control. Data are expressed as mean ± SD from three independent experiments. ### *p* < 0.001 compared with control group; * *p* < 0.05, ** *p* < 0.01, *** *p* < 0.001 compared with LPS alone-treated group

In general, the production of NO and PGE_2_ were related to the modulation of iNOS and COX-2 expression [19–21]. In view of obvious suppression of NO and PGE_2_ by PF, we also detected the protein levels of iNOS and COX-2 by Western blot analysis. As shown in Fig. 4, only a small amount of iNOS and COX-2 proteins were detected in unstimulated RAW264.7 macrophages; however, iNOS and COX-2 protein levels were considerably upregulated by LPS, and PF (3 μM, 6 μM and 12 μM) treatment all significantly reduced LPS-induced overexpression of iNOS and COX-2 protein in a dose-dependent manner; thereinto, low concentration (3 μM) treatment had a weak inhibitory effect on the iNOS and COX-2 protein levels (*p* < 0.05, *p* < 0.05), and highest concentration of 12 μM conspicuously decreased the production of iNOS and COX-2 by 87.6% and 68.2% respectively (*p* < 0.001, *p* < 0.001), compared with LPS alone-treated group. These results demonstrate that the inhibitory effects of PF on LPS-stimulated NO and PGE_2_ production are related to the suppression of iNOS and COX-2 expression.

### Inhibition of PF on NF-κB signaling pathway in LPS-stimulated RAW 264.7 macrophages

**Fig. 5:**
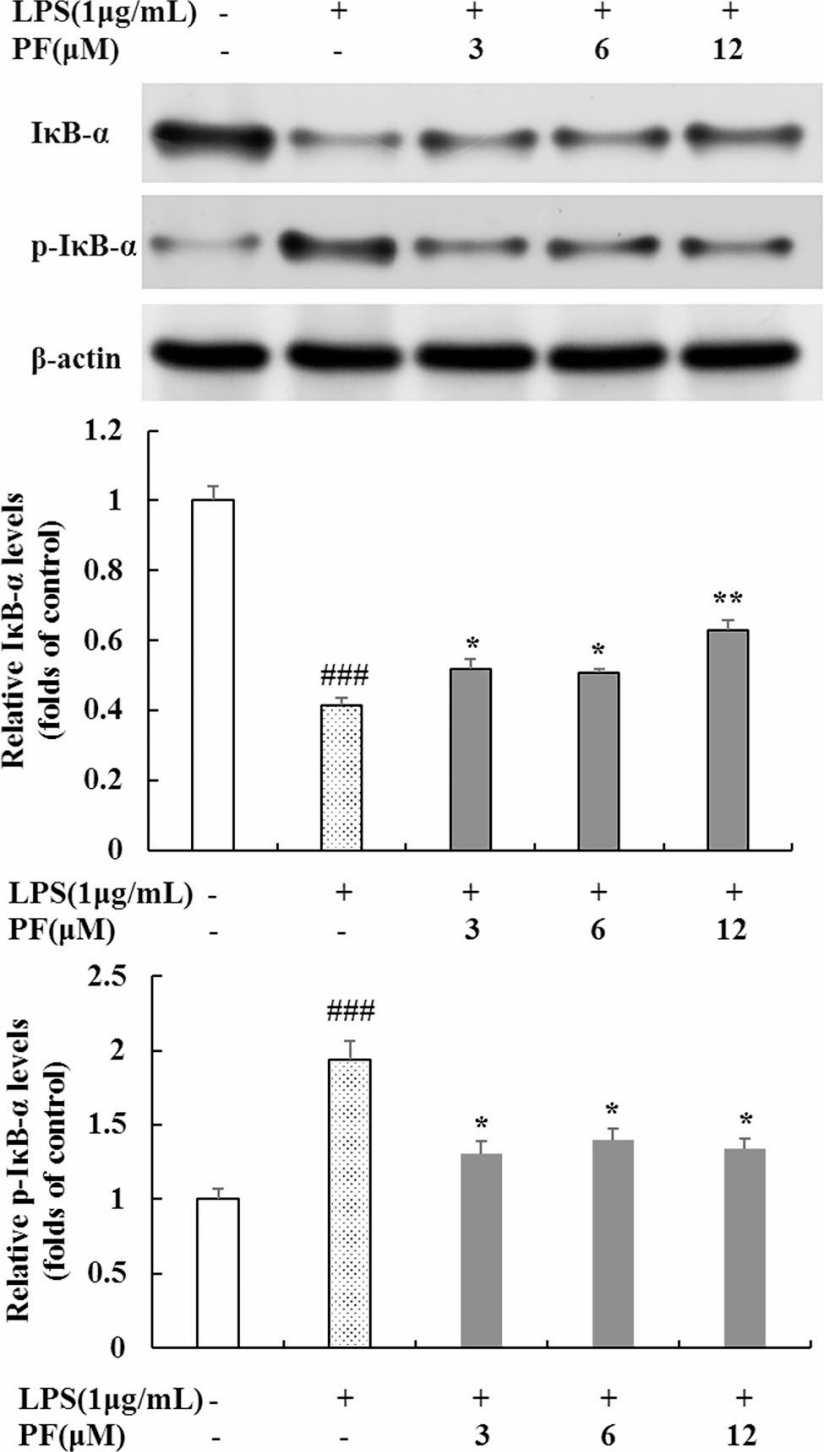
Effects of PF on IκB-α and p-IκB-α protein levels in LPS-stimulated RAW 264.7 macropages. RAW 264.7 cells were pretreated with PF (0, 3, 6, and 12 μM) for 1 h, followed by the addition of LPS (1 μg/mL) for 24 h. The protein levels of IκB-α and p-IκB-α in cytoplasmic proteins were examined by Western blot analysis. β-actin was used as a loading control. Neither LPS nor PF-treated group was selected as control. Data are expressed as mean ± SD from three independent experiments. ### *p* < 0.001 compared with control group; * *p* < 0.05, ** *p* < 0.01 compared with LPS alone-treated group

**Fig. 6:**
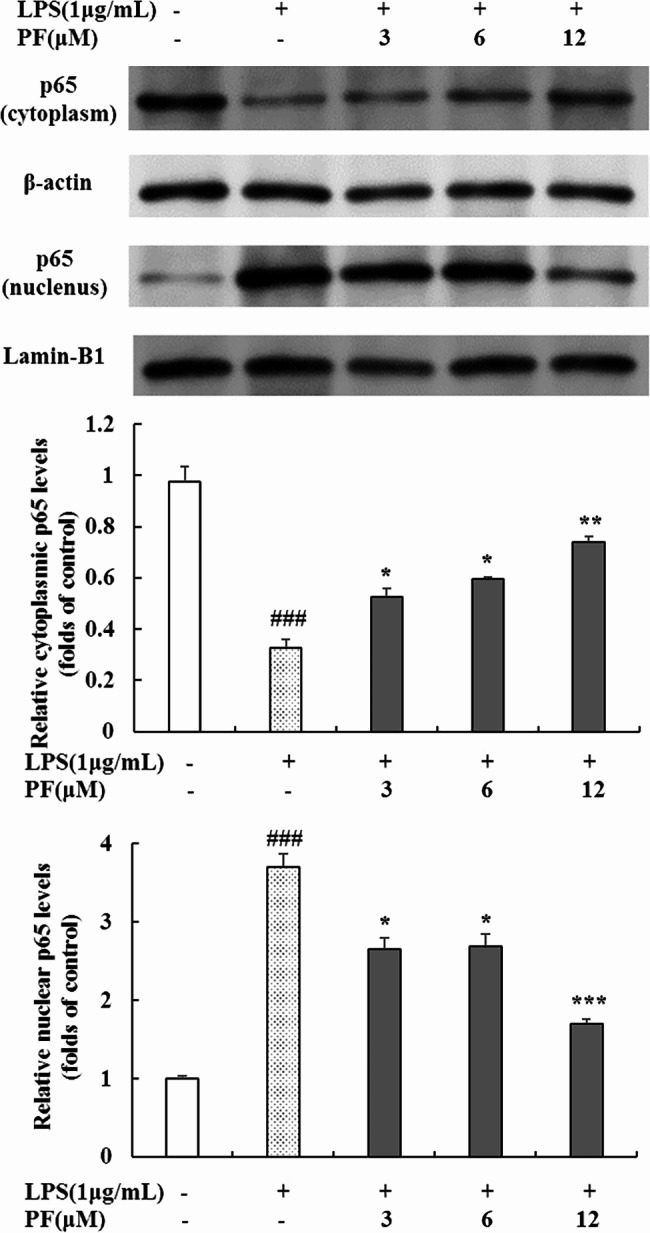
Effects of PF on cytoplasmic p65 and nuclear p65 protein levels in LPS-stimulated RAW 264.7 macropages. RAW 264.7 cells were pretreated with PF (0, 3, 6, and 12 μM) for 1 h, followed by the addition of LPS (1 μg/mL) for 24 h. The protein levels of cytoplasmic p65 and nuclear p65 were examined by Western blot analysis. β-actin and Lamin B1 were used as loading controls. Neither LPS nor PF-treated group was selected as control. Data are expressed as mean ± SD from three independent experiments. ## *p* < 0.01, ### *p* < 0.001 compared with control group; * *p* < 0.05, ** *p* < 0.01, *** *p* < 0.001 compared with LPS alone-treated group

Since NF-κB activation is critical for the LPS-stimulated activation of iNOS, COX-2, TNF-α, IL-1β and IL-6 [20–24], we monitored the protein expression of NF-κB signaling pathway including IκB-α, p-IκB-α and p65 by Western blot analysis. As shown in Fig. 5, PF (3 μM, 6 μM and 12 μM) inhibited the overexpression of p-IκB-α and reversed the degradation of IκB-α induced by LPS, whereas there was no significant diference in the inhibition effect of high concentration treatment and low concentration treatment. IκB-α phosphorylation and degradation could promote NF-κB translocation to the nucleus, therefore we further examined the nucleus translocation of p65, a major component of NF-κB. The results (Fig. 6) demonstrated that 3 μM, 6 μM and 12 μM of PF decreased the concentration of p65 in the nucleus in LPS‑stimulated RAW 264.7 macrophages (*P* < 0.05, *P* < 0.05, *P* < 0.001). In contrast, the expression level of p65 in the cytoplasm was upregulated by PF (*P* < 0.05, *P* < 0.05, *P* < 0.01). These results suggest that PF have a repressive effect on the LPS-stimulated NF-κB signaling pathway.

### Inhibition of PF on Akt phosphorylation in LPS-stimulated RAW 264.7 macrophages

**Fig. 7:**
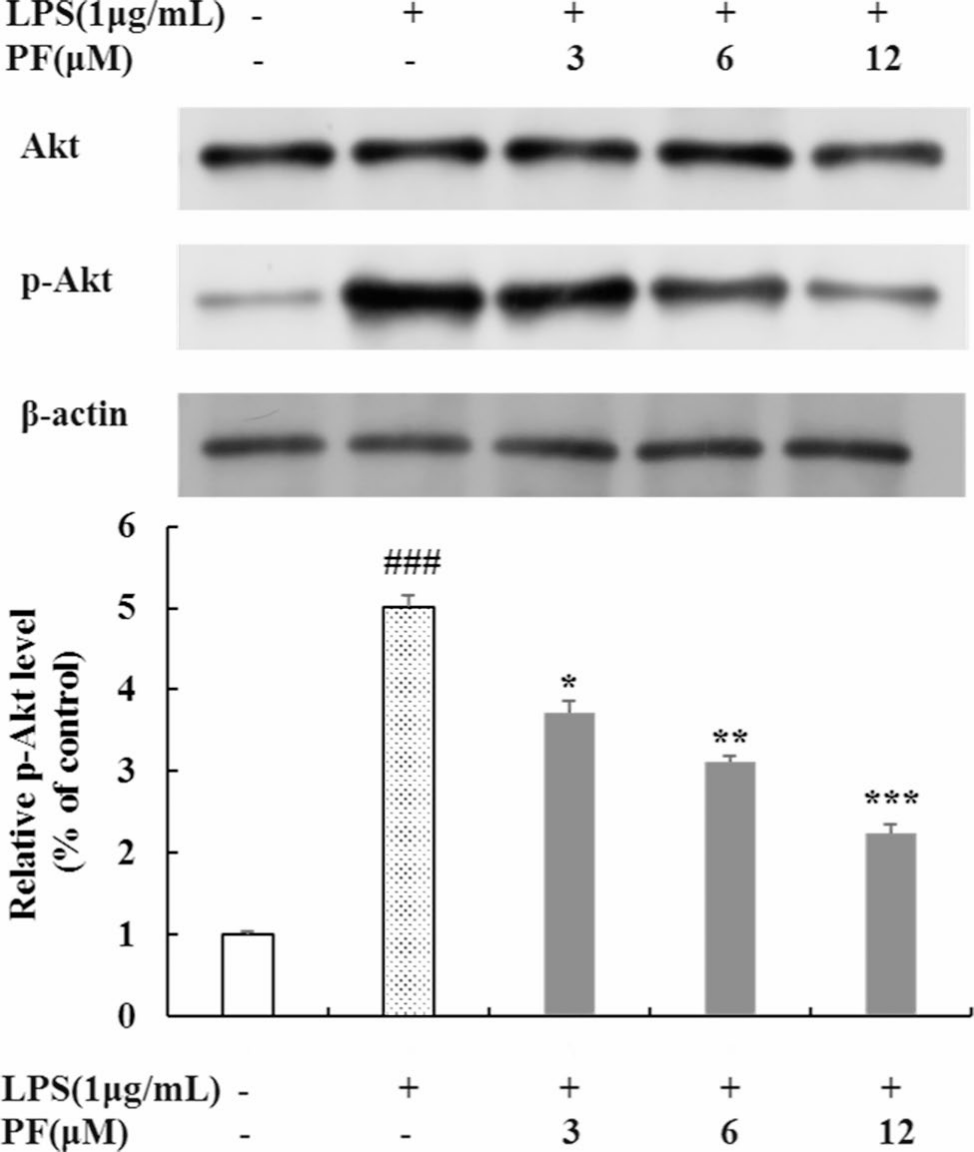
Effects of PF on phosphorylation level of Akt in LPS-stimulated RAW 264.7 macropages. RAW 264.7 cells were pretreated with PF (0, 3, 6, and 12 μM) for 1 h, followed by the addition of LPS (1 μg/mL) for 24 h. The protein levels of Akt and p-Akt in cytoplasmic proteins were examined by Western blot analysis. β-actin was used as a loading control. Neither LPS nor PF-treated group was selected as control. Data are expressed as mean ± SD from three independent experiments. ### *p* < 0.001 compared with untreated control group; * *p* < 0.05, ** *p* < 0.01, *** *p* < 0.001 compared with LPS alone-treated group

Akt signaling pathway plays a crucial role in the activation of NF-κB signaling pathway in inflammatory processes [8, 9, 19, 25]. Therefore, we continued to exam the effect of PF on Akt phosphorylation by Western blot analysis. As shown in Fig. 7, PF (3 μM, 6 μM and 12 μM) had no effect on non-phosphorylated Akt protein level, but it significantly and dose-dependently diminished the phosphorylation of Akt caused by LPS in RAW 264.7 macrophages (*P* < 0.05, *P* < 0.01, *P* < 0.001). Moreover, 12 μM of PF treatment possessed a 65.5% inhibition rate of Akt phosphorylation in contrast with LPS alone‑treated group. These results indicate that PF could block the LPS-stimulated Akt pathway signaling.

### Inhibition of PF on MAPK phosphorylation in LPS-stimulated RAW 264.7 macrophages

**Fig. 8:**
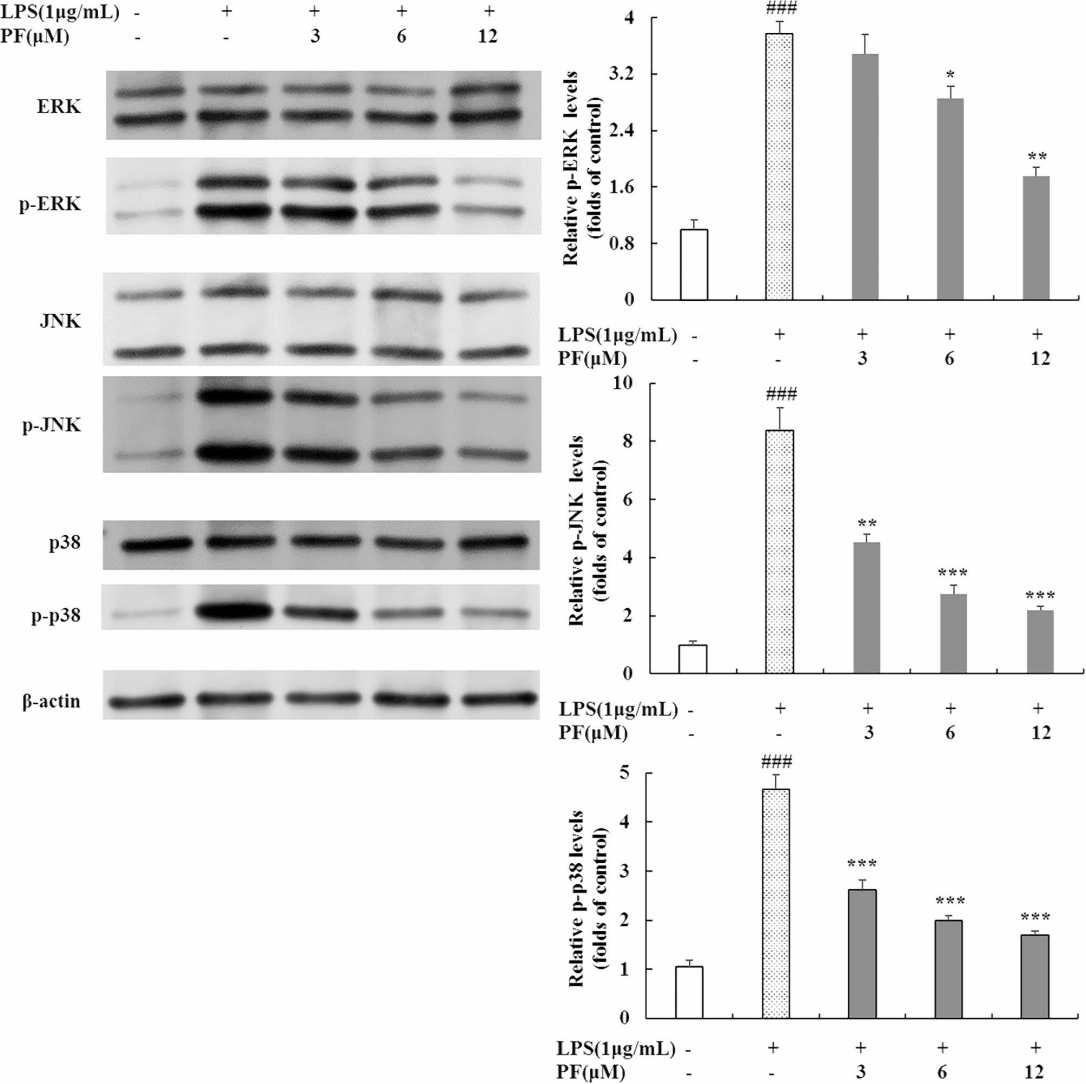
Effects of PF on phosphorylation levels of MAPK in LPS-stimulated RAW 264.7 macropages. RAW 264.7 cells were pretreated with PF (0, 3, 6, and 12 μM) for 1 h, followed by the addition of LPS (1 μg/mL) for 24 h. The protein levels of ERK/p-ERK, JNK/p-JNK and p38/p-p38 in cytoplasmic proteins were examined by Western blot analysis. β-actin was used as a loading control. Neither LPS nor PF-treated group was selected as control. Data are expressed as mean ± SD from three independent experiments. ### *p* < 0.001 compared with control group; * *p* < 0.05, ** *p* < 0.01, *** *p* < 0.001 compared with LPS alone-treated group

MAPK signaling pathways are closely related to the inflammatory response [8, 9, 19, 26, 27]. Therefore, we monitored the MAPK signaling pathways including ERK, JNK and p38 pathways by Western blot analysis. As shown in Fig. 8, the phosphorylation of ERK, JNK and p38 were in a low level in uninduced RAW264.7 macrophages, and those were markedly upregulated after LPS stimulation (*P* < 0.001, *P* < 0.001, *P* < 0.001). As expected, the expression of p‑ERK, p-JNK, and p-p38 were dose-dependently downregulated in the PF‑treated LPS‑stimulated RAW264.7 macrophages in contrast with LPS alone‑treated group (Fig. 8), and the expression level of those three phosphorylated proteins were declined by 53.6%, 73.8% and 62.4% with high concentration of PF treatment (12 μM) (*P* < 0.01, *P* < 0.001, *P* < 0.001). However, the expression of non-phosphorylated ERK, JNK and p38 was not interfered by LPS or LPS plus PF. These results imply that PF suppresses inflammatory cytokine expression by inhibiting the phosphorylation of MAPK signaling pathway.

## Discussion

An increasing number of research indicates that natural products obtained from herbs are becoming an invaluable source of anti-inflammatory drug research and development [28, 29]. In this aspect, NRR merits our attention because it has been widely used for thousands of years as a crucial traditional Chinese medicine for the treatment of inflammatory diseases [16]. In view of cumarins, such as notopterol, isoimperatorin and nodakenetin from NRR, exhibitting moderate anti-inflammatory effects [30], we has tried to find more potent constituents which are responsible for the anti-inflammatory actions of NRR. Consequently, we isolated PF, a phenolic acid ester, from NRR [17]; however, its anti-inflammatory effect and underlying mechanism of action have not been well elucidated. In the present study, we evaluated the anti-inflammatory properties and molecular mechanisms of PF in LPS-stimulated RAW 264.7 macrophages.

During the inflammatory process, an excess of inflammatory mediator and cytokines (e.g., NO, PGE_2_, TNF-α, IL-1β and IL-6) are released [2]. Macrophages play a crucial part in the regulation of inflammatory processes, and they will be activated to produce numerous inflammatory mediators and cytokines by LPS [1, 10, 11]. Therefore, LPS triggers the inflammatory response, and LPS-stimulated RAW 264.7 macrophages have been widely applied to explore the underlying mechanisms of new anti-inflammatory agents in vitro [10, 11]. In our previous study, we found that PF remarkably inhibited NO production in LPS-stimulated RAW 264.7 macrophages [17]. Continually, we focused on the effects of PF on the levels of PGE_2_, TNF-α, IL-1β, IL-6 and IL-10 production in this work. As a consequence, PF significantly inhibited the release of PGE_2_, TNF-α, IL-1β and IL-6 in LPS-induced macrophage activation (Figs. 2 and 3).

NO and PGE_2_, classic biomarkers of inflammatory reaction and crucial inflammatory mediators, are excessively secreted in LPS-induced RAW 264.7 macrophages, and as we know they are biosynthesized by the iNOS protein and COX-2 protein respectively [19, 20]. Therefore, we measured the influence of PF on iNOS protein and COX-2 protein using Western blot analysis so as to investigate its anti-inflammatory properties. As observed in our study, PF exhibited remarkable inhibitory effect on the LPS-induced upregulation of iNOS and COX-2 protein expression in a dose-dependent manner (Fig. 4). Based on these results, we conclude that PF reduces inflammation-mediated factors via downregulation of iNOS and COX-2 protein expression.

NF-κB is tightly involved in regulating inflammation as a central transcription factor responsible for the secretion of inflammatory mediators and cytokines (e.g., iNOS, COX-2) in LPS-stimulated RAW 264.7 macrophages. NF-κB consists of p50 and p65 subunits, which are present in the cytoplasm and bind to IκB in unstimulated macrophages. Upon exposure to LPS, NF-κB is activated, which promotes phosphorylation-induced proteasomal degradation of IκB from the IκB/NF-κB complex and facilitates NF-κB translocation to nucleus and its following inflammation-related genes transcriptions [20–24]. Based on the above evidence, we determined the influence of PF on protein expression of IκB-α, p-IκB-α and p65 aiming to explore its anti-inflammatory mechanisms. The results revealed that PF significantly attenuated the LPS-activated phosphorylation of IκB-α and prevented p65 translocation to the nucleus (Figs. 5 and 6), indicating that the anti-inflammatory effect of PF is related to the inhibition of NF-κB signaling pathway.

Numerous studies have demonstrated that the Akt signaling pathway has functions as a upstream molecule of the NF-κB activation [8, 9, 19, 25]. Akt can be activated by LPS-induced TLR4-mediated pathway, which plays a critical role in NF-κB activation and inflammation responses [8, 9, 25]. Therefore, we explored the effect of PF on Akt signaling in LPS-induced RAW264.7 macrophages. Our results reported that PF significantly weakened LPS-induced Akt phosphorylation (Fig. 7). Taken together, PF reduces LPS-induced inflammatory responses by downregulating the Akt-mediated NF-κB signaling pathway.

Besides NF-κB and Akt signaling pathways, MAPK, including ERK and two stress-activated protein kinase families, JNK and p38, are stimulated by TLR4 and play an important role in inflammatory response in macrophages [8, 9, 19, 26, 27]. Therefore, much interest had focused on the effects of PF on the phosphorylation of ERK, JNK and p38 in LPS-stimulated RAW 264.7 macrophages. In our study, PF significantly diminished LPS-induced phosphorylation of ERK, JNK and p38 in RAW264.7 cells (Fig. 8), implying that PF exerts anti-inflammatory activities through blocking MAPK signaling pathway.

## Conclusions

**Fig. 9:**
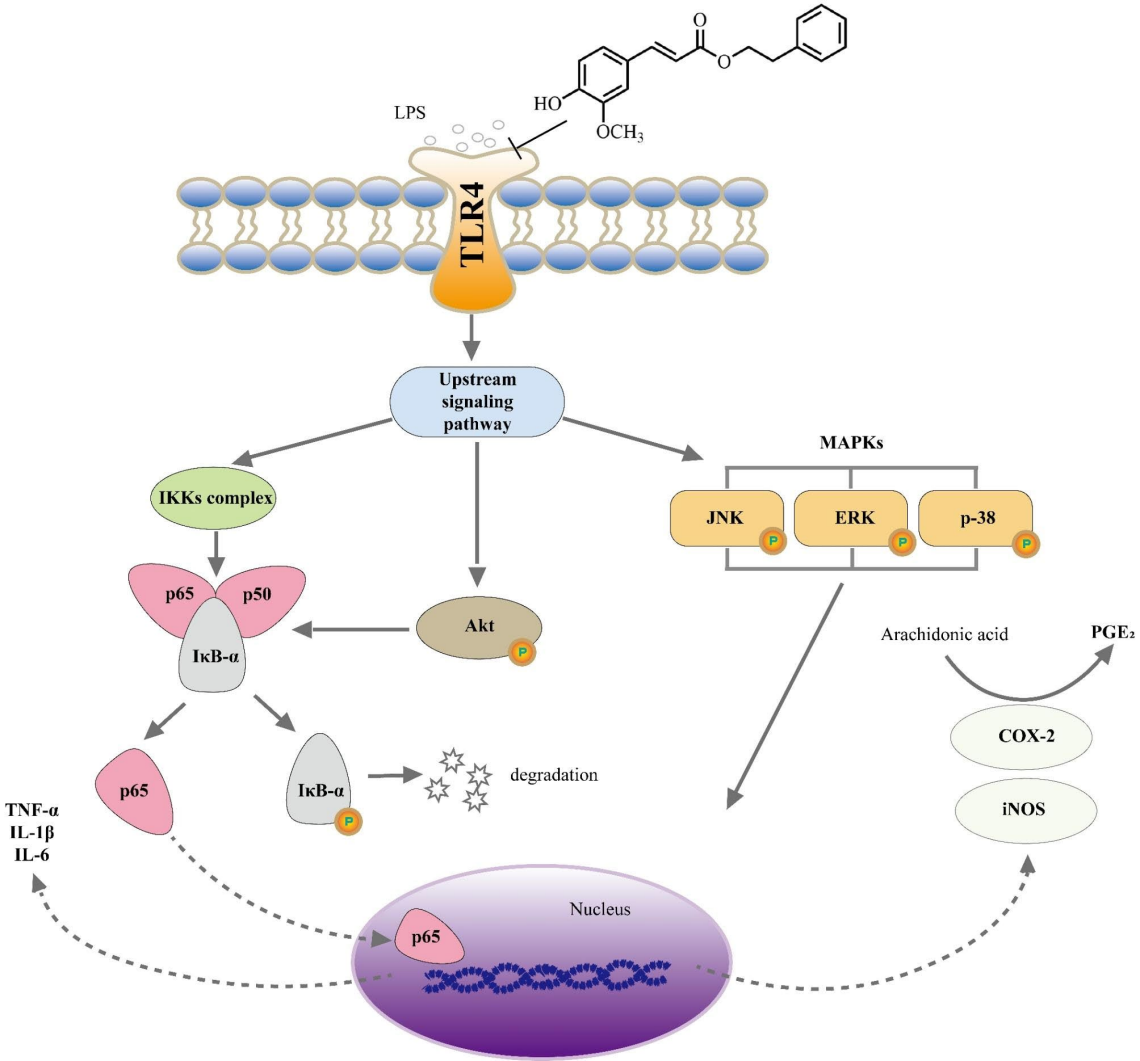
Schematic representation of the proposed anti-inflammatory mechanisms of PF in LPS-stimulated RAW 264.7 macrophages

In summary, PF, a natural phenolic acid ester obtained from NRR, downregulates the production of inflammatory mediators and cytokines, including NO, PGE_2_, TNF‑α, IL-1β, IL‑6, iNOS and COX-2 in LPS-stimulated RAW 264.7 macrophages. Intracellular signal transduction pathway study shows that the inhibition of PF on NF-κB is achieved via suppressing IκB-α phosphorylation and preventing p65 translocation to the nucleus, and it is activated by upstream Akt phosphorylation. Besides, the molecular anti-inflammatory mechanism of PF is also associated with inhibiting MAPK activation, which results from a blockade of JNK, ERK, and p38 phosphorylation(Fig. 9). The findings of the present study suggest that PF could be considered as a natural inhibitor of NF-κB, Akt and MAPK signaling pathways and it is expected to be a possible anti-inflammatory candidate. This study lay a foundation for exploring new anti-inflammatory targets so as to further elucidate the anti-inflammatory mechanism of PF at the cellular and molecular level; at the same time, it also provides a new perspective for the design of innovative anti-inflammation drugs with the characteristics of traditional Chinese medicine. Besides, further research is required to determine its anti-inflammatory properties in vivo.

### Electronic supplementary material

Below is the link to the electronic supplementary material.

Supplementary Material 1

## Data Availability

The datasets used and analysed during the current study available from the corresponding author on reasonable request.

## Abbreviations

Akt: Protein kinase B
BCA: Bicinchoninic acid
BSA: Bovine serum albumin
COX-2: Cyclooxygenase 2
DMEM: Dulbecco’s modified eagle’s medium
DMSO: Dimethyl sulfoxide
ECL: Enhanced chemilu-minescent
ELISA: Enzyme-linked immunosorbent assay
ERK: Extracellular signal-regulated kinase
FBS: Fetal bovine serum
IκB-α: Inhibitor of NF-κB kinase-α
IL-1β: Interleukin 1β
IL-6: Interleukin 6
iNOS: Inducible nitric oxide synthase
JNK: C-Jun N-terminal kinases
LPS: Lipopolysaccharide
MAPK: Mitogen-activated protein kinase
MTT: 3-(4,5-Dimethylthiazol-2-yl)-2,5-diphenyltetrazolium bromide
NF-κB: Nuclear factor kappa-B
NO: Nitric oxide
NRR: *Notopterygii Rhizoma et Radix*
PBS: Phosphate buffered saline
PF: Phenethylferulate
PGE_2_: Prostaglandin E_2_
RIPA: Radioimmunoprecipitation assay
TLRs: Toll-like receptors
TNF-α: Tumor necrosis factor-α
