# Supplementary material for: Phenethylferulate as a natural inhibitor of inflammation in LPS-stimulated RAW 264.7 macrophages: focus on NF-κB, Akt and MAPK signaling pathways
Source: BMC Complement Med Ther. 2023 Nov 7;23:398. doi: 10.1186/s12906-023-04234-y (PMC10629144; doi:10.1186/s12906-023-04234-y)
Supplement: Supplementary file 1 — Supplementary Material 1 [file 12906_2023_4234_MOESM1_ESM.pdf]

Additional file:

Phenethylferulate as a natural inhibitor of inflammation in LPS-stimulated RAW 264.7 macrophages: Focus on NF-κB, Akt and MAPK signaling pathways

Zhongjie Yan[1†], Yuanyu Wang[1†], Yizhen Song[2], Yicong Ma[2], Yufan An[2], Ran Wen[2], Na Wang[2], Yun Huang[2] and Xiuwen Wu[2*]

Some additions for the western blot experimental pictures are provided. This file contained as full as possible length gels and blots with membrane edges visible. These images were the original, unprocessed versions.

Groups notes are as follows:

Control group: Neither LPS nor PF-treated group

Model group: LPS alone-treated group

PF (3 μM) group: LPS plus PF (3 μM) treated group

PF (6 μM) group: LPS plus PF (6 μM) treated group

PF (12 μM) group: LPS plus PF (12 μM) treated group

Supplementary Figure 1 (additions for Figure 4 in the main text)

iNOS: 125KDa	COX-2: 72KDa	β-actin: 42KDa
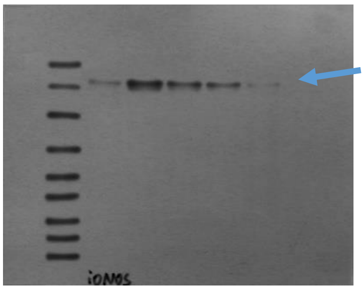	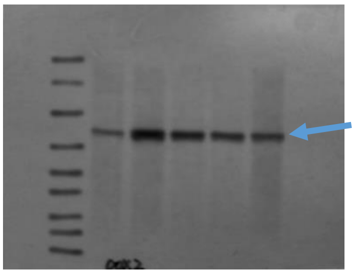	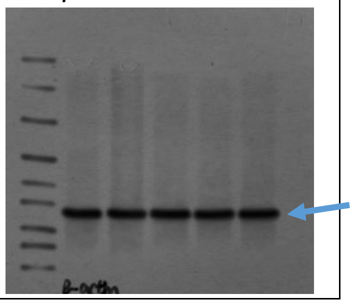

In each image, the lane from left to right is Marker, Control group, Model group, PF (3 μM) group, PF (6 μM) group and PF (12 μM) group. The arrow points to iNOS protein, COX-2 protein and β-actin respectively.

Supplementary Figure 2 (additions for Figure 5 in the main text)

IκB-α: 40 KDa	p-IκB-α:40 KDa	β-actin: 42 KDa
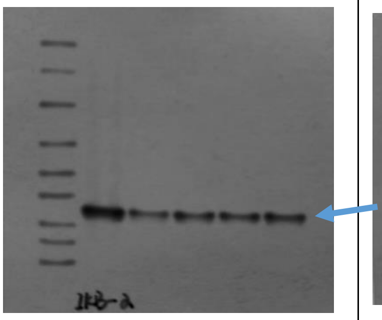	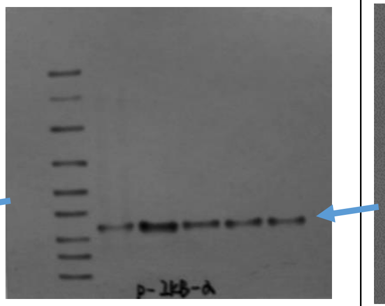	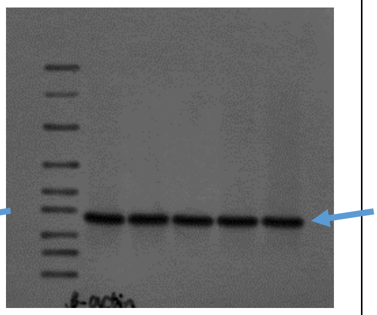

In each image, the lane from left to right is Marker, Control group, Model group, PF (3 μM) group, PF (6 μM) group and PF (12 μM) group. The arrow points to IκB-α protein, p-IκB-α protein and β-actin respectively.

Supplementary Figure 3 (additions for Figure 6 in the main text)

cytoplasmic p65: 65 KDa 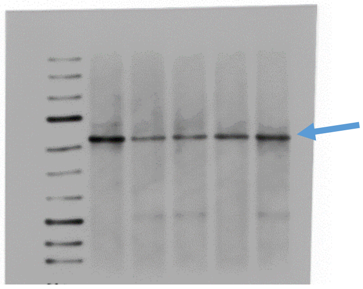	β-actin: 42 KDa 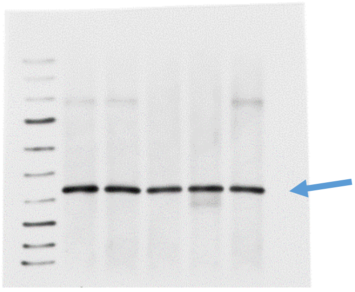
nuclear p65: 65 KDa 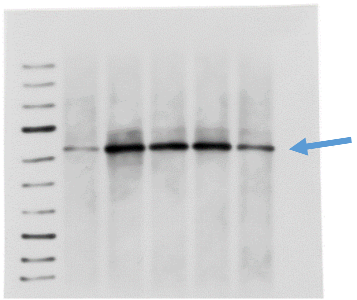	Lamin B1: 67 KDa 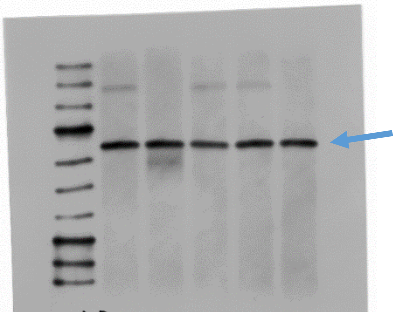

In each image, the lane from left to right is Marker, Control group, Model group, PF (3 μM) group, PF (6 μM) group and PF (12 μM) group. The arrow points to cytoplasmic p65 protein, β-actin, nuclear p65 protein and Lamin B1 respectively.

Supplementary Figure 4 (additions for Figure 7 in the main text)

Akt: 60 KDa	p-Akt: 60 KDa	β-actin: 42 KDa
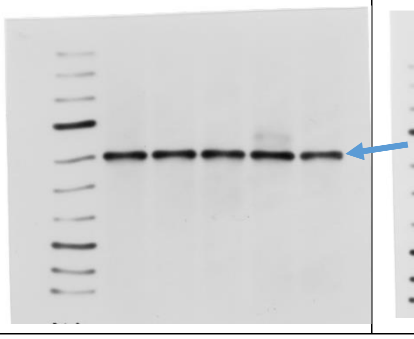	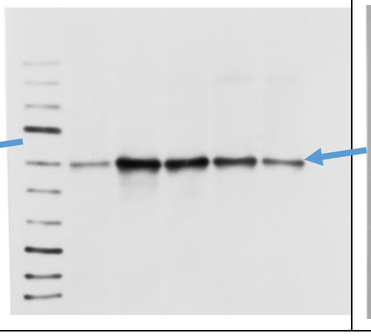	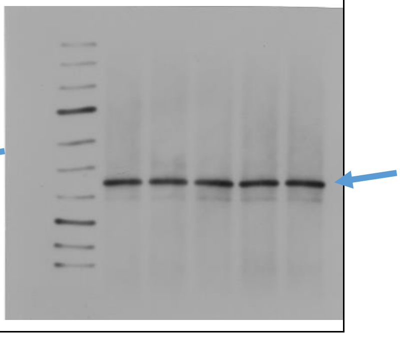

In each image, the lane from left to right is Marker, Control group, Model group, PF (3 μM) group, PF (6 μM) group and PF (12 μM) group. The arrow points to Akt protein, p-Akt protein, and β-actin respectively.

Supplementary Figure 5 (additions for Figure 8 in the main text)

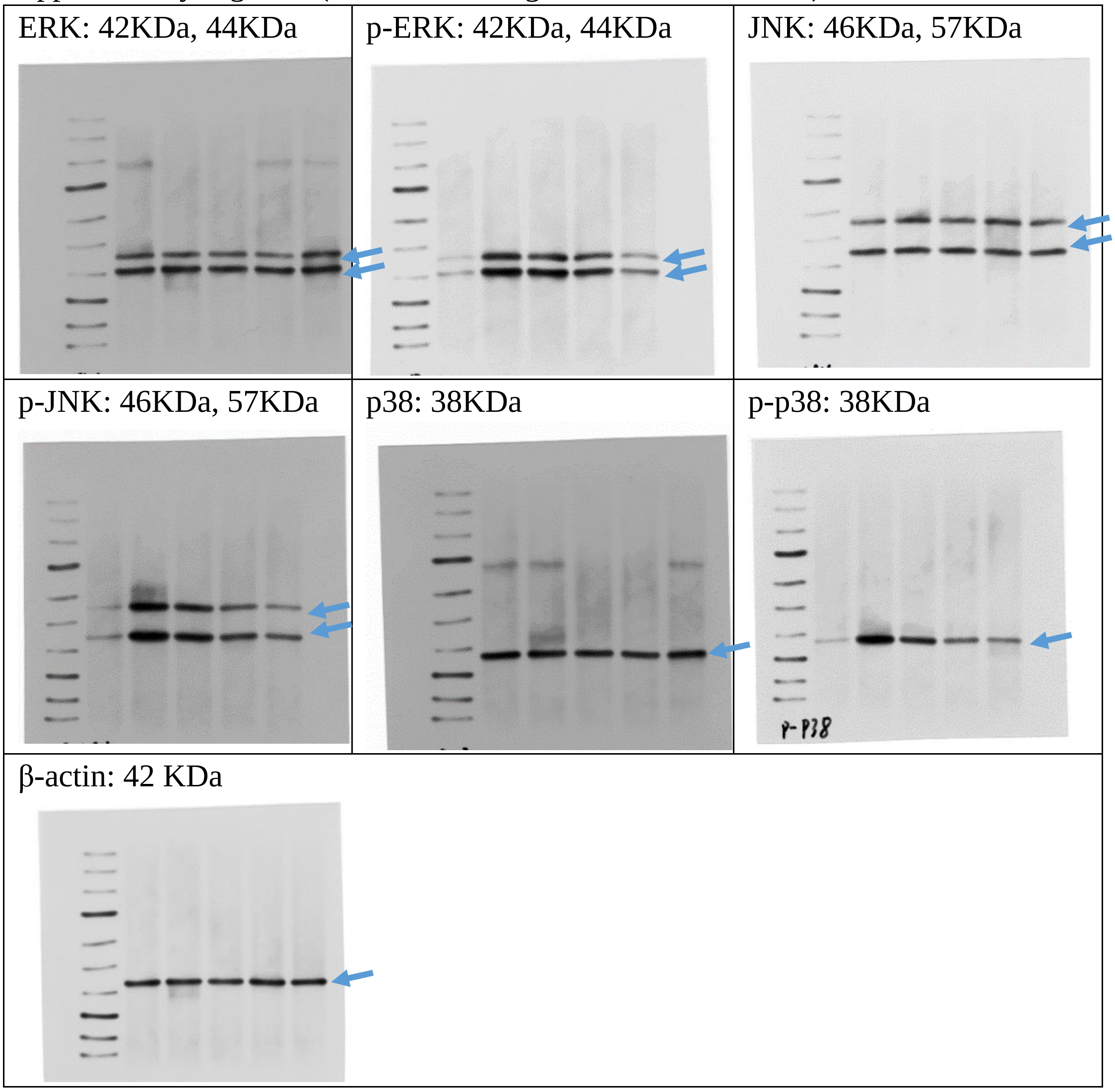

In each image, the lane from left to right is Marker, Control group, Model group, PF (3 μM) group, PF (6 μM) group and PF (12 μM) group. The arrow points to ERK protein, p-ERK protein, JNK protein, p-JNK protein, p38 protein, p-p38 protein and β-actin respectively.
